# Evaluation of serum antioxidant markers and calcium levels in women with Arthritis

**DOI:** 10.12669/pjms.42.8.14392

**Published:** 2026-08

**Authors:** Abdul Rehman, Muhammad Jamil, Maham Farhan, Amna Saleem Khan, Mahroo Javaid, Aqsa Iftikhar, Uzma Urooj Malik

**Affiliations:** 1 Abdul Rehman, PhD Scholar, Department of Biochemistry, University of Karachi, Karachi, Pakistan; 2 Muhammad Jamil, MBBS, FCPS, Pediatric Fellowship (Egypt) Assistant Professor, Orthopedic Surgery Department, Civil Hospital Karachi, Pakistan; 3 Maham Farhan, BS Biochemistry, Department of Biochemistry, University of Karachi, Karachi, Pakistan; 4 Amna Saleem Khan, BS Biochemistry, Department of Biochemistry, University of Karachi, Karachi, Pakistan; 5 Mahroo Javaid, MSc Biochemistry, Department of Biochemistry, University of Karachi, Karachi, Pakistan; 6 Aqsa Iftikhar, BS Biochemistry, Department of Biochemistry, University of Karachi, Karachi, Pakistan; 7 Uzma Urooj Malik, PhD Assistant Professor, Department of Biochemistry, University of Karachi, Karachi, Pakistan

**Keywords:** Osteoarthritis, Rheumatoid Arthritis, Oxidative Stress, Glutathione Reductase, Reduced Glutathione, Catalase, Superoxide dismutase, Calcium

## Abstract

**Background & Objective:**

Osteoarthritis (OA) and Rheumatoid Arthritis (RA) are chronic, degenerative and inflammatory joint diseases, aggravated by reactive oxygen species (ROS) and impaired antioxidant defense system. However, gender based oxidative stress analysis remain under-examined, despite increasing evidence points towards sex-based biochemical changes in arthritis patients. The study aimed to measure the antioxidant activities of enzymes including superoxide dismutase (SOD), Glutathione reductase (GR) alongside reduced glutathione (GSH), and catalase (CAT) in female arthritis patients. Calcium balance is also estimated in order to analyze the bone demineralization.

**Methodology:**

The case-control study was designed and conducted at the Orthopedic Department of a Dr. Ruth K. M. Pfau, Civil hospital, Karachi. Serum levels of GSH, GR, SOD, CAT, and calcium were evaluated in 42 arthritis patients and 16 age matched healthy female controls by using standard spectrophotometric and biochemical assays. Data was expressed as mean ± SD and analyzed statistically to compare both the groups.

**Results:**

Arthritis patients in comparison with healthy controls showed significant decrease in GSH (p < 0.001) and GR (p < 0.01) levels, while SOD and CAT levels were slightly reduced but non-significant. Similarly, slightly higher calcium levels were also observed, suggesting altered mineral homeostasis associated with inflammatory conditions. The simultaneous decrease in antioxidant enzyme activities suggests the impaired redox homeostasis in female patients.

**Conclusion:**

The study highlights an altered antioxidant status and disturbed calcium homeostasis in female patients. These alterations in biochemical system suggest the therapeutic potential of antioxidant and calcium modulating interventions as effective treatment for managing and slowing disease progression in females.

## INTRODUCTION

Arthritis is the most common and prevalent joint disease characterized by degenerative and inflammatory changes in joints. This disease affects millions of people worldwide, and is associated with pain, swelling, stiffness and reduced mobility. There are many forms of arthritis, but osteoarthritis (OA) and rheumatoid arthritis (RA) are the most prevalent types. OA is characterized by degeneration of articular cartilage, formation of osteophyte, subchondral bone sclerosis and inflammation of synovium.^1^ These structural changes contribute to functional impairment, joint pain, and stiffness more particularly in knees, hip joint and spine. In contrast, RA is chronic autoimmune disorder that primarily affects the joints but other organs could also be affected with RA. It is an autoimmune disease characterized by inflammation of synovium, formation of pannus that progressively destruct cartilage and bones.^2^ Although etiology and clinical sign of OA and RA differ fundamentally, both ultimately leads to joint destruction, deformity of bones, functional impairment and reduced quality of life. Despite OA and RA has different origin — mechanical and autoimmune, research suggests that these conditions become similar at molecular level during later stages of disease progression. Both undergo through inflammation and oxidative damage which progressively damage joints over time.^3^

Oxidative stress is one of the major contributor to joint damage in OA and RA. It arises from the disturbance between the production of reactive oxygen species (ROS) and the ability of body to neutralize it with antioxidants.^4^ Increased level of ROS which include, superoxide anions, hydroxyl radicals and peroxides can seriously cause the damage to biomolecules like membrane lipids, proteins and DNA. This damage can trigger NF-κB signaling pathway which upregulates the pro-inflammatory mediators like TNF- α, IL-6 and IL-1β.^5^ Activation of these inflammatory mediators enhance oxidative stress and destruction of joint tissues.^6^

Several studies have shown that the joint tissues and synovial fluid in patient of OA and RA contains high levels of oxidative damage products and low levels of defensive antioxidants.^6^,^7^ This oxidative imbalance not only increase inflammation but is also responsible for activation of matrix metalloproteinases (MMPs), promoting apoptosis of chondrocyte and inability of body to repair joints, which ultimately lead to cartilage matrix degradation.^8^ Therefore, studying oxidative damage and response of antioxidant is very important to understand the progression of disease in OA and RA. The antioxidant defense system consists of enzymatic and non-enzymatic components that neutralize the harmful effect of ROS. Major antioxidant enzymes include catalase (CAT), which decomposes hydrogen peroxide into water and oxygen; superoxide dismutase (SOD), which convert superoxide radicals into less reactive molecules and glutathione reductase (GR) enzyme which is responsible for maintaining the ratio between oxidized and reduced glutathione by converting oxidized glutathione (GSSG) into reduced glutathione (GSH). The reduced glutathione is a major form of antioxidant that directly neutralize the ROS.^9^ Furthermore, calcium a key mineral plays vital role in signal transduction pathway and inflammation. The imbalance of calcium homeostasis may contribute to oxidative damage in this disease.^10^ Altered levels of these biomarker indicates the oxidative state of the tissues and may serves as potential biomarkers in OA and RA patients.

Although, numerous studies on oxidative stress in arthritis were carried out, but very few of them have compared OA and RA in relation to antioxidant markers and calcium homeostasis particularly in women. Most previous studies included mixed gender population, which may mask biochemical differences that specifically affect women.^11^ Studies focused on female-based groups remain limited, despite evidences suggests that oxidative stress, inflammatory responses and calcium levels vary in female arthritis patients compared to males. Females are more commonly diagnosed with arthritis and are likely to experience more severe progression of disease compared to male, showing profound biological variations between both sexes. Hormones, specifically estrogen, further mediate antioxidant activity of enzyme and immunological reactions, making females clearly more susceptible to oxidative stress-linked joint destruction.^12^,^13^ The current study compares and evaluates antioxidant markers including SOD, GR, GSH, CAT, and calcium in female patients with OA and RA, to decipher their potential role in disease progression and underlying mechanisms.

## METHODOLOGY

The current study was designed as case-control study and conducted at the orthopedic department of a Dr. Ruth K. M. Pfau, Civil hospital, Karachi. All participants (Female subjects and healthy controls) were registered for multiple studies on arthritis after obtaining informed consent. A total of 42 samples from patients diagnosed with osteoarthritis and rheumatoid arthritis were collected over a period of three months. Among these, 36 patients were diagnosed with OA and 6 with RA. Clinical examination, MRI and X-ray imaging were employed to diagnose OA, while physical evaluation, blood tests and imaging were used for RA. Despite having mechanistic differences, both diseases share increased oxidative stress and diminished antioxidant defenses, justifying their grouped assessment.^14^ Every arthritis patient was prescribed with standard pharmacological medication by their physician.

### Ethical Approval:

Approval of current study was obtained by Institutional Bio-Ethical committee, University of Karachi (Approval No. IBC KU-507/2024; Dated: 5^th^ December 2024).

The criteria for inclusion and exclusion were set based on the demographic data and clinical characteristics. For osteoarthritis, inclusion of age specific criteria was also considered. Those patients who had a history of alcoholism, hypertension, thyroid dysfunction, diabetes mellitus, joint injuries, kidney and liver disease, coronary artery disease, abnormal blood pressure and history of anti-inflammatory medication usage were excluded from the study. For comparison, 16 age and sex-matched controls were taken for the study who had no history or complain with joints related conditions. The limited no of control group (n=16) shows a recruitment limitation; however, smaller control group size has been noticed in comparable biochemical analyses of arthritis.^5^

### Sample Collection:

Five ml of venous blood was drawn from each subject and control during morning time in clot activator tubes. After clot formation at room temperature, samples were centrifuged at 2500 rpm for 10 minutes. Resulting serum was collected and stored at -20 for further biochemical analysis. Samples showing visible signs of hemolysis or insufficient volume were excluded to ensure accuracy of data.

### Biochemical Estimations:

### Reduced Glutathione (GSH):

The levels of GSH in serum were measured by enzymatic recycling method as reported in Nature Protocols.^15^ This assay is based on the reaction between GSH and DTNB [5,5’-Dithio-bis-(2-nitrobenzoic acid)] in the presence of Glutathione Reductase and NADPH to form yellow color product called TNB (5-thio-2-nitrobenzoic acid). The change in absorbance was measured at 412 nm by using microplate reader.

### Glutathione Reductase (GR):

The enzyme activity of GR was measured by using oxidized glutathione as a substrate. The rate of NADPH consumption in the presence of oxidized glutathione is directly proportional to GR activity. The change in absorbance was monitored at 340nm.

### Catalase (CAT):

The activity of catalase was measured by observing the breakdown of hydrogen peroxide (H_2_O_2_) at 240nm. The reaction mixture contained Serum sample, phosphate buffer and hydrogen peroxide as the substrate.

### Superoxide Dismutase (SOD):

IFCC (International Federation of Clinical Chemistry) approved colorimetric kit (Cat. No. EIASODC, by Thermo Fisher Scientific) was used to measure the activity of SOD according to manufacturer’s instructions. The change in absorbance was measured at 450nm on a microplate reader.

### Calcium:

Colorimetric assay kit was used to measure the serum calcium which is based on o-cresolphthalein complexone method (instructed by Randox Laboratories, Manual RX Monza). Calcium ions react with o-cresolphthalein complexone in an alkaline medium to produce purple colored-complex which was measured at 570nm.

### Statistical Analysis:

Statistical analysis was done using Microsoft excel, SPSS, and Graph pad prism V.10.5. Data were expressed as mean ± standard deviation (SD). Normality was analyzed using Shapiro-Wilk test before assessment. Student’s t-test was employed and for non-normally distributed variables, Mann-Whitney U test was used as a non-parametric alternative. A p-value < 0.05 was considered significant statically.

## RESULTS

**Fig.1:**
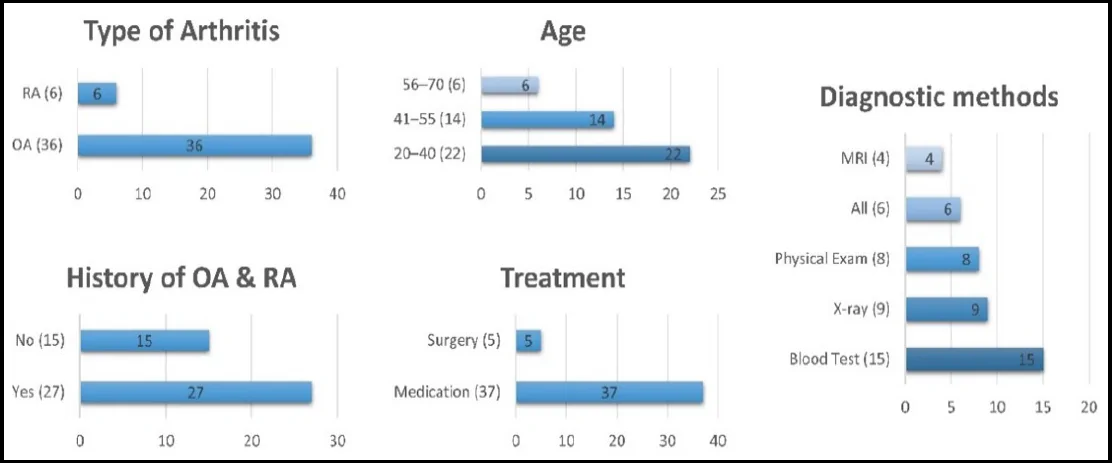
Demographic details of female subjects with Arthritis.

A total of 42 female arthritis patients with a mean age of 45.2 ± 11.3 years participated in the current study. The demographic characteristics which include the age, type of arthritis, history of OA and RA, diagnostic methods, and plan of treatment of the study participants are summarized in Fig. 1.]

### Reduced Glutathione (GSH):

**Fig.2:**
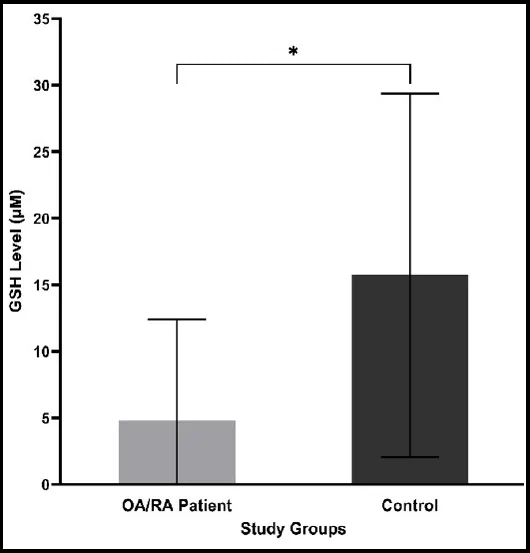
Comparison of serum Reduced Glutathione levels between arthritis patients and healthy controls (Mean ± SD).

Serum GSH levels were found to be decreased significantly in OA and RA patients compared to healthy controls (p < 0.001). The mean concentration of GSH was 4.78 ± 7.64 μM in the patient group, whereas the control group showed a mean concentration of 15.72 ± 13.66 μM, (Fig.2).

### Glutathione Reductase (GR) Activity:

**Fig.3:**
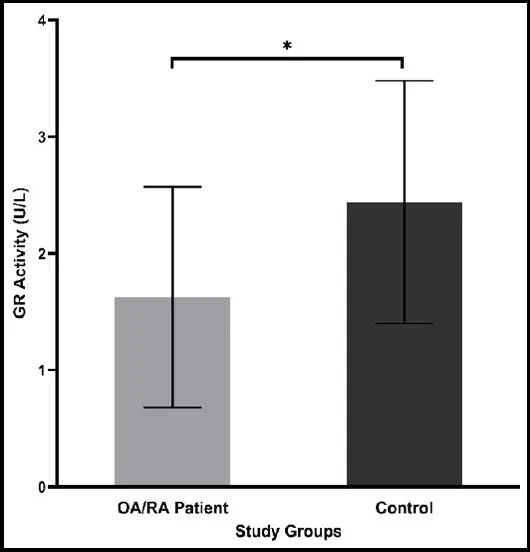
Comparison of Glutathione reductase activity between patients and controls (Mean ± SD).

A decrease in activity of GR was observed in OA and RA patients compared to healthy individual’s (p < 0.01). The mean activity of GR was 1.63 ± 0.95 U/L in the patient group, whereas mean of GR activity in control group was found to be 2.44 ± 1.04 U/L, (Fig.3).

### Catalase (CAT) Enzyme Activity:

**Fig.4:**
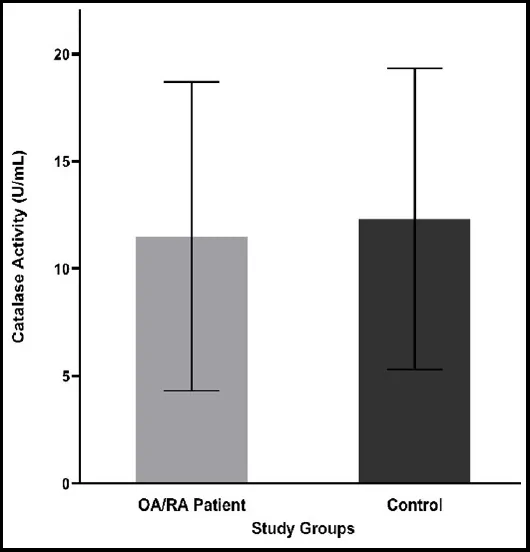
Comparison of Catalase activity between patients and controls (Mean ± SD).

Slightly lower mean activity of catalase was observed in OA and RA patients (11.52 ± 7.19 KU/L) compared to healthy individual’s (12.32 ± 7.01 KU/L). However, the difference was not significant (p > 0.05) as mentioned in Fig.4.

### Superoxide Dismutase (SOD) Activity:

**Fig.5:**
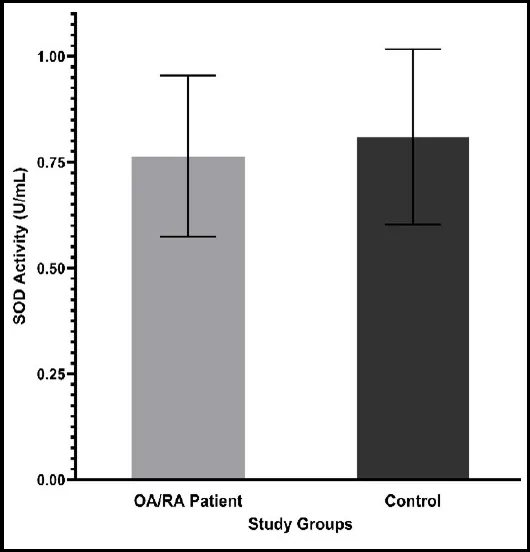
Comparison of Superoxide dismutase Levels between patients and controls (Mean ± SD).

Slightly lower mean activity of SOD was observed in OA and RA patients (0.76 ± 0.19 U/mL) compared to healthy individual’s (0.81 ± 0.21 U/mL). Although, the difference was not significant (p > 0.05) as illustrated in Fig.5.

### Calcium Levels:

**Fig.6:**
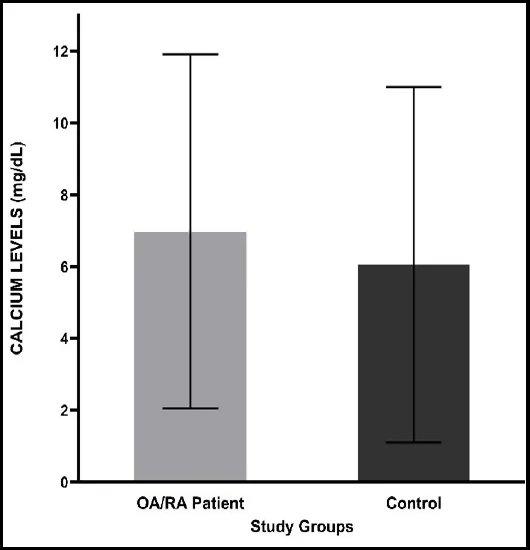
Comparison of Calcium Levels between patients and controls (Mean ± SD).

Slightly higher levels of calcium were observed in OA and RA patients compared to healthy individual’s (p> 0.05). Mean concentration of calcium was 6.98 ± 4.93 mg/dL in the patient group, whereas mean of control group has found 6.05 ± 4.95 mg/dL, as shown in Fig.6.

## DISCUSSION

In the present study, antioxidant markers were assessed in female arthritis patients to find the status of oxidative system and altered metabolic conditions. The antioxidant biomarkers including GSH and GR were significantly lower in arthritis patients compared to control group (p<0.01 and p<0.001 respectively), whereas SOD, CAT and serum calcium exhibited non-significant results. Arthritis covering OA and RA are the most common chronic joint diseases that progressively destruct articular cartilage, induce pain and loss of mobility function in patient. Oxidative stress play key role in the pathophysiology of both conditions by inducing cell damage through weak antioxidant defense machinery and increased production of ROS.^6^,^16^

Glutathione system based on reduced glutathione, is non-enzymatic molecule responsible for antioxidant activity by directly neutralizing free radicals and balancing redox system. This study showed significant reduced levels of GSH in OA and RA patients compared to healthy controls, suggesting reduced ROS detoxification and increased oxidative damage.^7^ Reduced level of GSH is likely due to its increased utilization during ROS neutralization or due to limited production of GSH from GSSG, ultimately showing compromised antioxidant capacity in Arthritis patients.^17^

GR is an enzyme which is responsible for converting GSSG into GSH by using NADPH.^18^ The enzyme activity of GR was also found significantly lower in arthritis patients compared to controls the reduced activity of GR impairs GSH recycling further contributing to oxidative stress mediated cellular damage.^14^ The simultaneous decrease of both GSH and GR levels indicates disruption in the glutathione antioxidant system in arthritis patients, which contributes to increased chronic inflammation and progressive joint damage in arthritis patients.^19^

Superoxide dismutase (SOD) and catalase (CAT) are important enzymatic antioxidants involved in the detoxification of reactive oxygen species. SOD catalyzes the conversion of superoxide radicals into O_2_ and H_2_O_2_. Slightly lower and non-significant activity of SOD was observed in patient with arthritis suggests impaired detoxification of superoxide radicals, thereby promoting oxidative damage and activating pro inflammatory pathways.^20^ Similarly CAT, an enzyme responsible for catalyzing hydrogen peroxide conversion into water and oxygen, was also found slightly reduced in arthritis patients. This indicates that the antioxidant machinery cannot effectively neutralize hydrogen peroxide produced by SOD which further promote oxidative damage and additional injury of tissues.^21^ Although, the reduction in the activity of both these enzymes SOD and CAT highlights a disruption in redox balance in patients with arthritis, further studies with large sample size are required to confirm these observations to establish their clinical relevance.

Calcium is an essential mineral that is important in maintaining and building bones and works as secondary messenger in variety of processes such as, immunological signaling and inflammatory cascades. The slightly elevated serum calcium levels in arthritis patients may be associated with bone demineralization due to chronic joint inflammation. Chronic inflammation promotes the secretion of pro-inflammatory cytokines, which trigger osteoclasts, leading to elevated bone resorption and subsequent calcium release into the blood circulation.^22^

Additionally, impaired homeostasis of calcium is associated with degradation of cartilage through activation of matrix metalloproteinases, which are responsible for breaking down extracellular matrix in arthritis.^23^ This supports the role of altered metabolism of calcium in the progression of joint deterioration and triggering of inflammatory cascades in patients with arthritis.^24^

Overall, the findings of the present study indicate an imbalance in antioxidant and metabolic biomarkers among female arthritis patients. Significant reduction in GSH levels and GR activity with slightly reduced SOD, CAT activity alongside increased levels of calcium, support the involvement of oxidative stress and altered metabolic regulation in arthritis progression. These findings are consistent with previous studies reporting impaired antioxidant defense mechanisms in arthritis patients.^5^,^18^,^25^ The study particularly focused on female patients and highlights the importance for gender specific investigations as both males and females have different oxidative and metabolic responses in disease. This study supports the idea of gender-specific research, to better understand the role of oxidative stress biomarkers in female arthritis patients and to explore the potential therapeutic value of antioxidant-based interventions.

### Limitations:

Although the study presents certain limitations such as unequal number of OA and RA patients, small number of control samples, and large standard deviation for GSH, further elucidation pertaining large sample size and within group comparison of OA and RA in future cohorts might be crucial in consolidating the outcomes.

## CONCLUSION

This study demonstrates altered antioxidant defense system and calcium homeostasis in female patients with arthritis. The reduction in the levels of GSH, GR, SOD and CAT suggests impaired antioxidant capacity increased oxidative stress which contributes to chronic joint inflammation and tissue damage. Simultaneously, increased levels of calcium may also reflect raised bone demineralization and possible mineral imbalance associated with disease pathology. Collectively, these findings indicate the oxidative stress and metabolic dysfunction in arthritis progression, supporting the therapeutic potential of antioxidant and calcium modulating interventions in the management of female arthritis patients.

### Author’s Contribution:

**AR:** Concept, study design, sample collection, data analysis, critical review and manuscript writing.

**UUM:** Concept, study design, data analysis, critical review and proofread the manuscript; corresponding author.

**MJ, AS, MF, MJv & AI:** Data collection, analysis and interpretation.

All authors are responsible and accountable for the integrity of the work and have read and approved the final manuscript.
